# Anogenital distance on MRI does not correlate to surgical diagnosis of endometriosis in patients without prior abdominal surgery

**DOI:** 10.1038/s41598-024-82407-6

**Published:** 2024-12-16

**Authors:** Sebastian Harth, Lena Metze, Daniel Leufkens, Fritz C. Roller, Alexander Brose, Felix Zeppernick, Ivo Meinhold-Heerlein, Gabriele A. Krombach

**Affiliations:** 1 Department of Diagnostic and Interventional Radiology, University Hospital Gießen, Justus Liebig University of Gießen, Klinikstr. 33, 35392 Giessen, Germany; 2 Department of Medicine, Institute of Medical Informatics, Justus Liebig University of Gießen, Rudolf-Buchheim-Straße 6, 35392 Giessen, Germany; 3 Department of Gynecology and Obstetrics, University Hospital Gießen, Justus Liebig University of Gießen, Klinikstr. 33, 35392 Giessen, Germany

**Keywords:** Endometriosis, Biomarkers, Diagnostic imaging, Magnetic resonance imaging, Pelvic Pain, Reproductive disorders, Urogenital diseases

## Abstract

Anogenital distance (AGD) is regarded as a potential biomarker for endometriosis, and a measurement on MRI images has been found to be promising. This study aimed to evaluate the measurement of AGD on MRI to predict the surgical diagnosis of endometriosis. We included 127 patients who received an MRI for endometriosis between October 2018 and February 2023. AGD was measured on MRI by two readers (MRI-AGD-AC: clitoris to anus; MRI-AGD-AF: posterior fourchette to anus). The feasibility and interobserver reliability of AGD measurements were evaluated. Differences in AGD between patient groups were analyzed. The intraclass correlation coefficient estimates indicated a good to excellent reliability of MRI-AGD-AC (0.92; 95% CI: 0.83–0.95) and a poor to good reliability of MRI-AGD-AF (0.68; 95% CI: 0.27–0.83). No statistically significant differences in the mean MRI-AGD-AC and MRI-AGD-AF in patients with and without surgical diagnosis of DIE (*p* = 0.413; *p* = 0.110), peritoneal endometriosis with and without DIE (*p* = 0.641; *p* = 0.323), and ovarian endometriosis (*p* = 0.155; *p* = 0.150) were found. The AUC ranged from 0.475 (95% CI: 0.365–0.584) to 0.586 (95% CI: 0.454–0.718). Thus, AGD does not constitute a valuable biomarker for patients with clinically suspected endometriosis.

## Introduction

Anogenital distance (AGD) is the distance between the anus and the genital tubercle that is present during the development of the reproductive system. Animal and human studies have shown that AGD is dependent on the early prenatal hormonal environment^1,2^. A longer AGD is considered to be a consequence of androgen exposure, and a shorter AGD a consequence of estrogen exposure. The AGD is regarded as a potential biomarker for various urogenital diseases, including hypospadias, prostate cancer, polycystic ovary syndrome (PCOS), and endometriosis^3^. As there currently is no test that can definitively confirm or rule out endometriosis non-invasively (although imaging is considered valuable^4–6^), the identification of biomarkers for this common disease is of interest^7^, with recent advances in the utilization of a saliva-based micro-ribonucleic acid (miRNA) signature for endometriosis^8^. About 10–15% of women of childbearing age are affected by endometriosis^9^. Several studies have shown promising results in predicting endometriosis based on AGD^10–13^, utilizing two clinical measurements: From the upper verge of the anus to the anterior surface of the clitoris (AGD-AC) and to the posterior fourchette (AGD-AF). Crestani et al. found that measurement of the AGD on MRI is more accurate than clinical measurement, and they reported significantly different values for AGD in endometriosis (*n* = 67) and non-endometriosis (*n* = 31) groups^14^. Conversely, Buggio et al. found no significant association between clinical AGD measurements and the presence of endometriosis in a case-control study, including 45 patients with deep infiltrating endometriosis (DIE), 45 patients with ovarian endometriomas and 45 controls^15^. Although all patients in the study by Crestani et al. underwent surgery, the patients in the control group were operated on for various different reasons (e.g., myomectomies, sacrocolpopexies), resulting in heterogeneity between the endometriosis group and the non-endometriosis group^14^. In the study by Buggio et al., the control group also came from a different population than the disease groups (e.g., periodic well-woman visits, cervical cancer screening)^15^.

Since it is necessary to validate a supposed biomarker on a study population that reflects the target population in which it will be used, we conducted a study on a collective of patients who had not undergone prior abdominal surgery, had an MRI scan for endometriosis and had subsequently undergone surgery within a maximum of one year of the MRI examination. This study aimed to compare measurements of MRI-AGD-AC and MRI-AGD-AF between patients with and without a subsequent surgical diagnosis of DIE, peritoneal endometriosis with and without DIE, and ovarian endometriosis (endometriomas). The value of AGD for the prediction of endometriosis was to be evaluated.

## Methods

This study was approved by the Ethics Committee of the Faculty of Medicine, Justus Liebig University Giessen (reference: 209/22). Informed consent was waived for this retrospective analysis by the Ethics Committee of the Faculty of Medicine, Justus-Liebig-University Giessen. All research was performed in accordance with relevant guidelines and regulations.

### Study population and design

All patients aged at least 18 years who had received a pelvic MRI for endometriosis between October 2018 and February 2023 were consecutively included. Patients who had not undergone surgery within one year of the MRI examination (as the disease status might have changed over an extended period^16^), patients with prior abdominal surgeries, and patients with insufficient coverage of the sagittal T2 sequence were excluded.

MRI scans were conducted at two 1.5 Tesla scanners (*n* = 70; *n* = 56) and one 3 Tesla scanner (*n* = 1). The MRI protocol included T2-weighted FSE (fast spin echo) sequences of the pelvis (axial, sagittal, coronal) as recommended in current guidelines^17,18^. The further sequences acquired were not relevant for the evaluation presented here. Patient preparation included rectal opacification with water (*n* = 121) and vaginal opacification with ultrasound gel (*n* = 107). These measures are considered optional or recommended conditionally in current guidelines and are performed by many institutions^17,18^. A shift of the anatomical landmarks relevant for the measurement of AGD is not to be expected by vaginal gel or rectal water given the rigid fibrous skeleton constituted by the interposed perineal body. Nevertheless, significant differences between the measurements performed on examinations with and without vaginal opacification have been excluded, as described in the results section.

### Image analysis

**Fig. 1:**
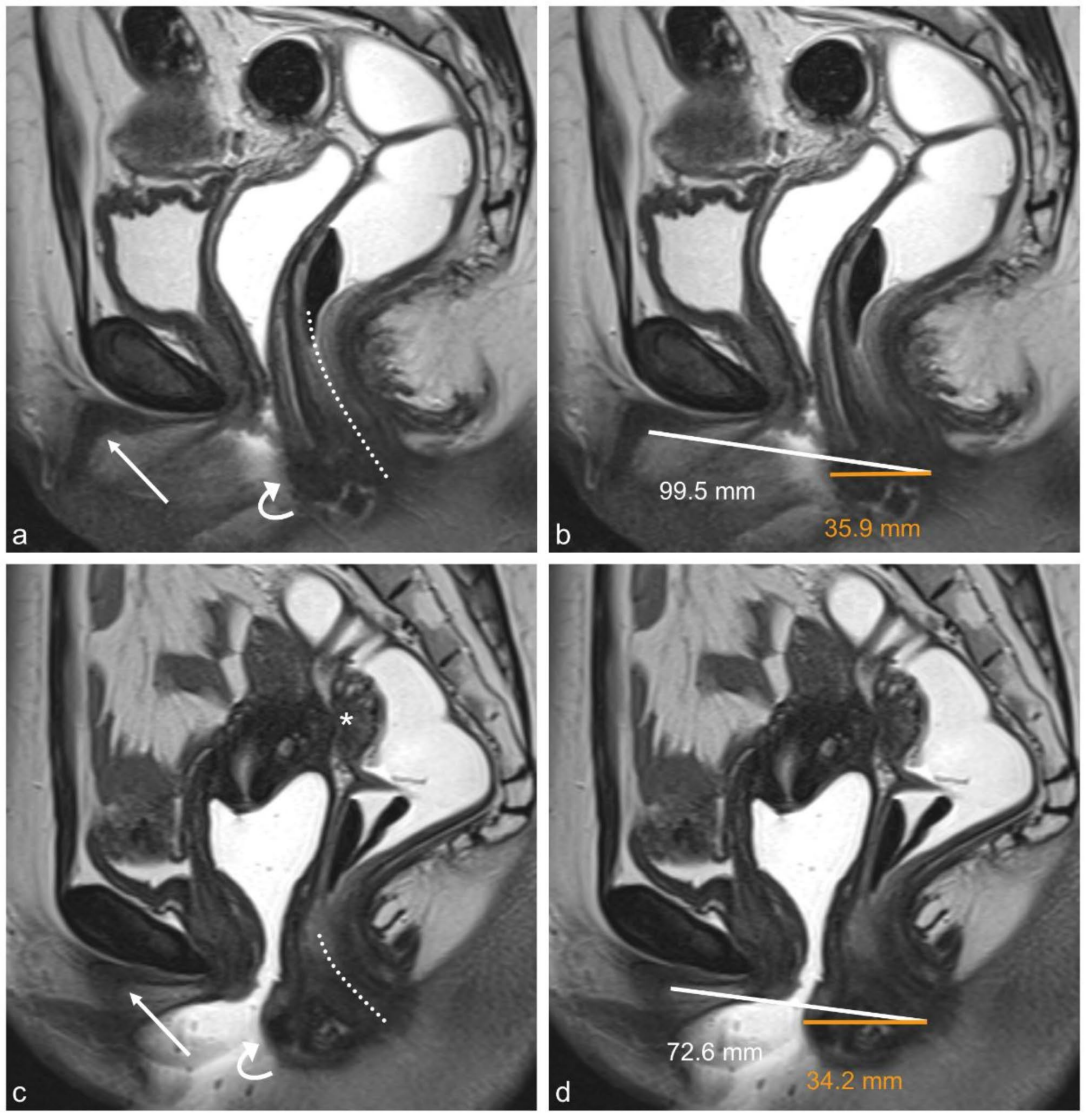
Two examples of anogenital distance (AGD) measurements in a 35-year-old woman without deep infiltrating endometriosis (DIE) (**a**, **b**) and a 31-year-old woman with DIE (**c**, **d**) on sagittal T2-weighted FSE (fast spin echo) sequences. **a**,** c** Anatomical landmarks for the measurements are the lower edge of the midline of the anal canal (dotted line), the posterior fourchette (curved arrow), and the posterior wall of the clitoris (straight arrow). **b**,** d** MRI-AGD-AC measurements from the posterior wall of the clitoris to the lower edge of the anal canal giving distances of 99.5 mm and 72.6 mm, respectively (white lines and numbers); MRI-AGD-AF measurements from the posterior fourchette to the lower edge of the anal canal giving distances of 35.9 mm and 34.2 mm, respectively (orange lines and numbers). Asterisk in **c**: Typical DIE with involvement of the rectum and the uterine cervix.

AGD measurements were performed independently by a reader with eight years of experience in pelvic MRI (reader 1, radiologist) and a reader not experienced in pelvic MRI (reader 2, advanced medical student). Reader 2 received a detailed tutorial on how to identify the relevant anatomical structures on T2-weighted images of the pelvis by reader 1 in advance. Measurements of AGD were performed on sagittal T2-weighted images from the anus to the clitoris (MRI-AGD-AC) and from the anus to the posterior fourchette (MRI-AGD-AF) as demonstrated in Fig. 1.

The center of the lower edge of the anal canal (i.e., the anus) served as posterior reference point for the measurements due to the favorable reproducibility, like Liu et al. described for clinical AGD measurements^19^. To record potential difficulties with the AGD measurements, readers noted measurability for each examination using a two-point scale.

### Surgical procedures

Surgeries were performed by senior surgeons with at least 10 years’ experience in endometriosis surgery, certified for minimally invasive surgery by the German Gynecological Endoscopy Working Group (AGE). The attending Department of Gynecology and Obstetrics is a training center for minimally invasive surgery, certified by the German Gynecological Endoscopy Working Group and by EuroEndoCert on behalf of the German Endometriosis Research Foundation (level III endometriosis center). Detection of DIE, peritoneal endometriosis with and without DIE, and ovarian endometriosis was noted as recorded in the surgery reports (laparoscopy, *n* = 123; laparotomy, *n* = 4).

### Statistical analysis

Statistical analyses were performed using IBM SPSS Statistics 29.0 (IBM), R 4.3.2 (The R Foundation for Statistical Computing), and RStudio 2023.12.1 + 402 (Posit Software). Continuous variables are presented as mean and standard deviation and categorical variables are presented as counts and percentages. The chi-square test and Fisher’s exact test were used to compare differences in the frequencies of categorical variables. Intraclass correlation coefficient (ICC) estimates were calculated for the AGD measurements of the readers, based on a mean-rating (k = 2), absolute-agreement, 2-way mixed-effects model and interpreted as suggested by Koo and Li^20^. Mean values of the measurements of MRI-AGD-AC and MRI-AGD-AF of the two readers were calculated for each patient. The Shapiro-Wilk test was used to test if the AGD measurements (MRI-AGD-AC, MRI-AGD-AF) were normally distributed in the group of patients with and without a surgical diagnosis of DIE. The Wilcoxon test (i.e., Mann-Whitney U test/ Wilcoxon rank-sum test) was then performed to assess differences in the mean AGD measurements between readers and patient groups. Binary logistic regression models were calculated to investigate the dependence between age, body mass index (BMI), MRI-AGD-AC, MRI-AGD-AF, and the variables “DIE”, “peritoneal endometriosis with and without DIE” and “ovarian endometriosis”. The sample size was based on the common requirement of a minimum number of events per variable of ten. The area under the receiver operating characteristic (ROC) curve (AUC) was calculated as a measure of the ability to discriminate the presence of DIE, peritoneal endometriosis with and without DIE, and ovarian endometriosis. P-values ≤ 0.05 were considered statistically significant.

## Results

**Fig. 2:**
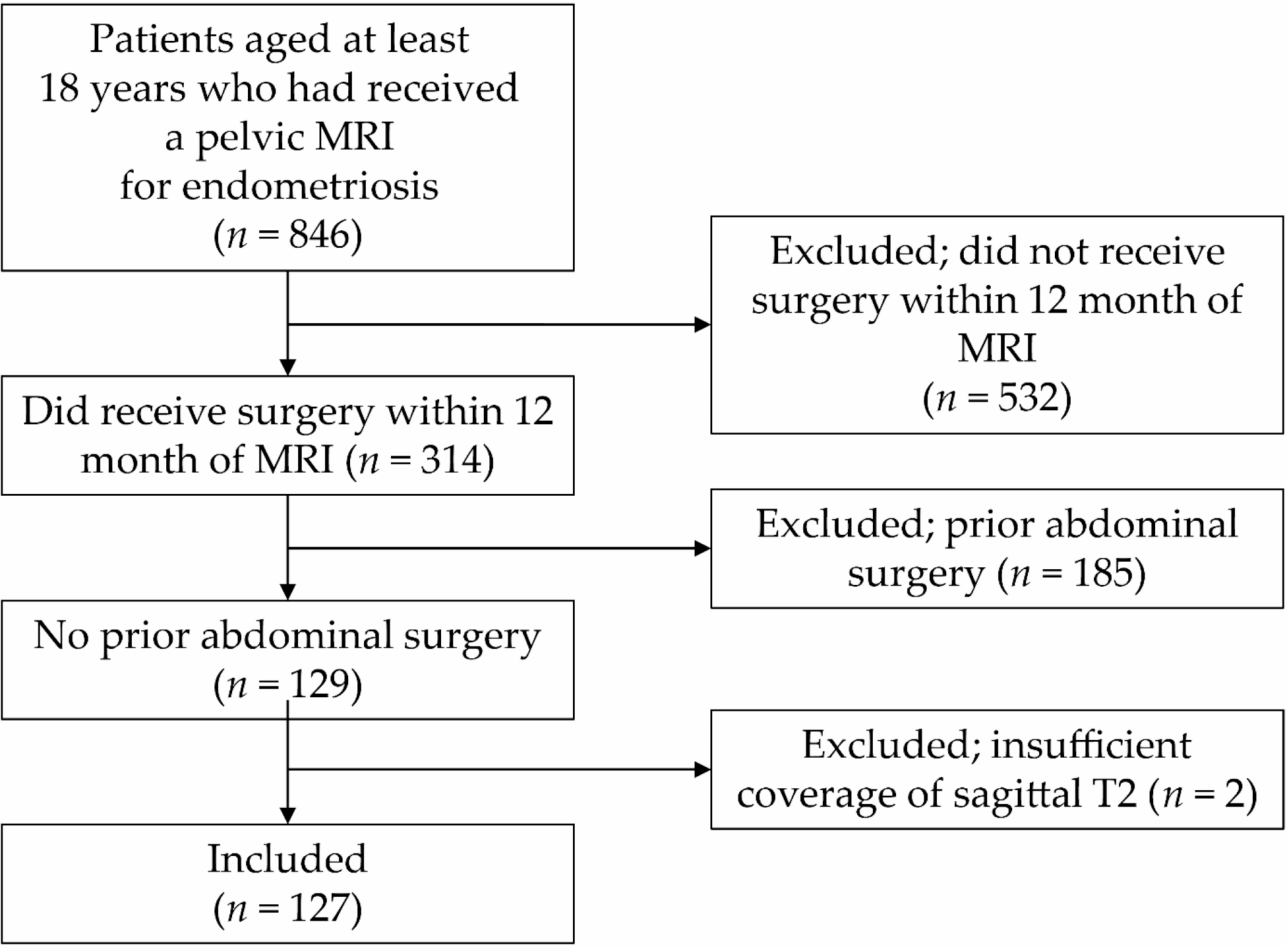
Flowchart of the study, demonstrating the study cohort.

127 consecutive patients (mean age, 30.4 years ± 6.7 [standard deviation]) were evaluated. The flowchart of our study cohort is provided in Fig. 2.

Characteristics of the study population are summarized in Table 1.

**Table 1:** Patient demographics and clinical characteristics.

	*N*/total (%) unless shown otherwise			
Characteristic	All patients (*n* = 127)	Surgery: DIE (*n* = 67)	Surgery: No DIE (*n* = 60)	*p*-value
Age (years), mean ± SD, range	30.4 ± 6.7, 18–49	30.9 ± 6.1	29.8 ± 7.3	0.199^a^
BMI (kg/m^2^) ± SD	24.5 ± 6.0	25.4 ± 6.8	23.6 ± 4.8	0.187^a^
Clinical symptoms				
Chronic pelvic pain	112/127 (88.2)	59/67 (88.1)	53/60 (88.3)	0.962^b^
Dysmenorrhea	90/127 (70.9)	46/67 (68.7)	44/60 (73.3)	0.563^b^
Dyspareunia	44/127 (34.6)	21/67 (31.3)	23/60 (38.3)	0.409^b^
Dyschezia	30/127 (23.6)	20/67 (29.9)	10/60 (16.7)	0.081^b^
Infertility	30/127 (23.6)	22/67 (32.8)	8/60 (13.3)	**0.010** ^**b**^
Abnormal uterine bleeding	21/127 (16.5)	10/67 (14.9)	11/60 (18.3)	0.606^b^
Dysuria	18/127 (14.2)	9/67 (13.4)	9/60 (15.0)	0.800^b^
Diarrhea/obstipation	11/127 (8.7)	9/67 (13.4)	2/60 (3.3)	**0.043** ^**b**^
Rectal bleeding	1/127 (0.8)	0/67 (0.0)	1/60 (1.7)	0.472^c^

DIE, deep-infiltrating endometriosis; ^a^ Wilcoxon test; ^b^ Chi-square-test; ^c^ Fisher’s exact text; bold values denote statistical significance at the *p* ≤ 0.05 level.

Cases and controls of the primary categories of the examination (surgical detection of DIE, *n* = 67 / no surgical detection of DIE, *n* = 60) had similar demographic and clinical characteristics. Only the symptoms infertility and diarrhea/obstipation were significantly more frequent in patients with subsequent surgical diagnosis of DIE (*p* = 0.010 and *p* = 0.043).

Reader 1 noted good measurability of AGD in 93/127 (73.2%) cases and limited measurability in 34/127 (26.8%) cases. Reader 2 noted good measurability of AGD in 57/127 (44.9%) cases and limited measurability in 70/127 (55.1%) cases. These differences in the categorizations of the measurability of AGD by the two readers were statistically significant (*p* < 0.001, chi-square test).

**Fig. 3:**
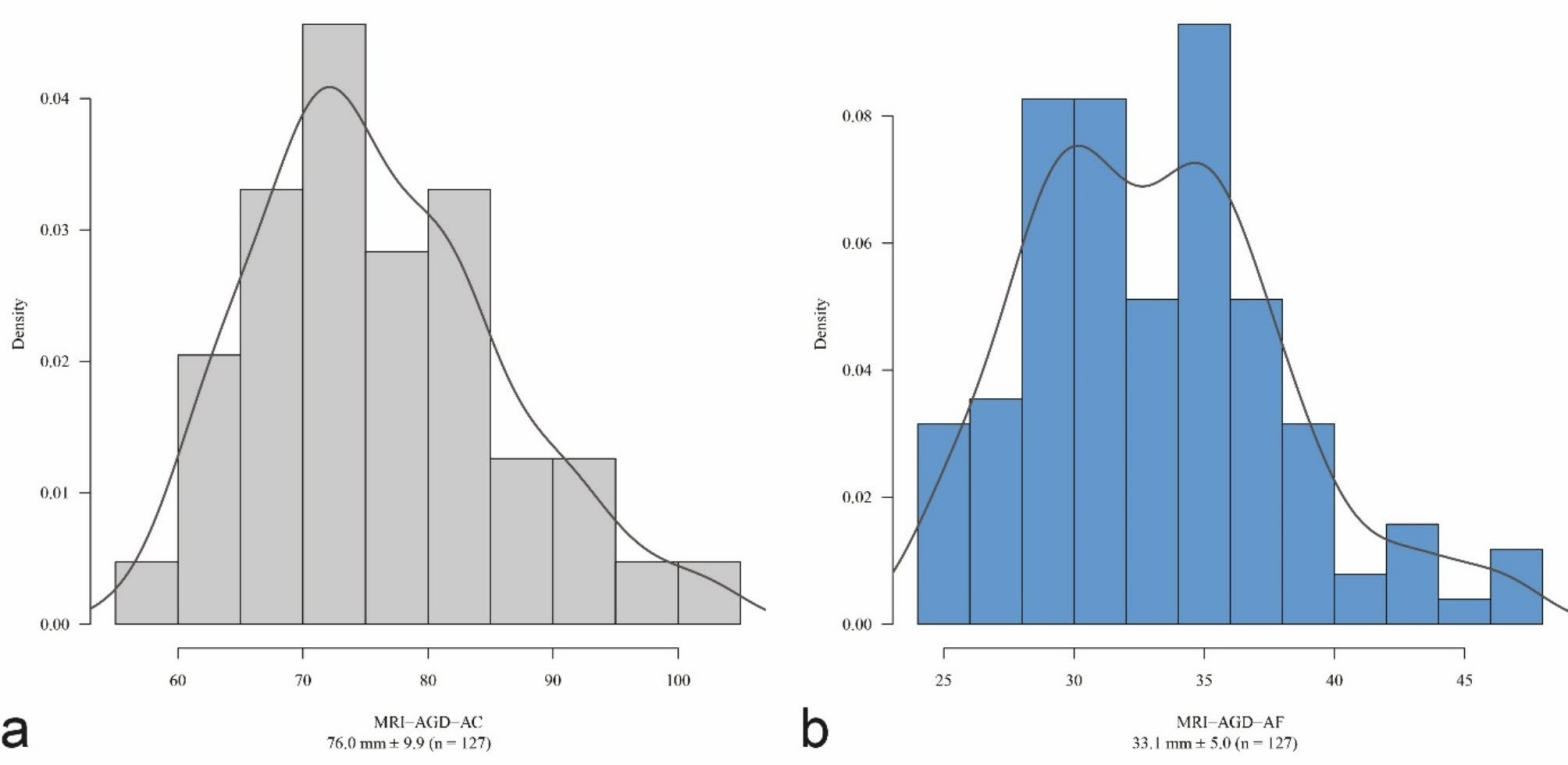
Distribution of the MRI-AGD-AC (**a**) and the MRI-AGD-AF (**b**) in the study population.

The mean values of the MRI-AGD-AC were 74.7 mm ± 10.4 (reader 1) and 77.4 mm ± 10.1 (reader 2) (*p* = 0.034, Wilcoxon test). The mean values of the MRI-AGD-AF were 31.3 mm ± 5.6 (reader 1) and 34.9 mm ± 5.4 (reader 2) (*p* < 0.001, Wilcoxon test). The averaged mean values were 76.0 mm ± 9.9 (reader 1 + 2) for MRI-AGD-AC, and 33.1 mm ± 5.0 (reader 1 + 2) for MRI-AGD-AF. Given the statistically significantly different mean values of AGD-measurements of both readers, analyses were performed for the AGD measurements of each reader individually and for averaged measurements. Figure 3 shows the frequency distribution of the averaged values of the AGD measurements of readers 1 and 2.

The ICC estimate of MRI-AGD-AC was 0.92 (95% CI: 0.83–0.95), indicating good to excellent reliability. The ICC estimate of MRI-AGD-AF was 0.68 (95% CI: 0.27–0.83), indicating poor to good reliability. When considering only the measurements for which good measurability was documented by both readers (*n* = 53), the ICC estimate of MRI-AGD-AC was 0.96 (95% CI: 0.90–0.98), indicating excellent reliability, and the ICC estimate of MRI-AGD-AF was 0.73 (95% CI: 0.43–0.86), indicating poor to good reliability.

The respective mean MRI-AGD-AC and MRI-AGD-AF were 76.1 mm ± 9.8 and 33.1 mm ± 4.8 in examinations with vaginal opacification (*n* = 107), and 75.9 mm ± 10.9 and 33.6 mm ± 6.0 in examinations without vaginal opacification (*n* = 20). No statistically significant differences between the measurements performed on examinations with and without vaginal opacification could be found (*p* = 0.784 and *p* = 0.686, respectively, Wilcoxon test).

The Shapiro-Wilk test for MRI-AGD-AC and MRI-AGD-AF did show a significant deviation from normal distribution for patients without a surgical diagnosis of DIE (*p* = 0.013 and *p* = 0.009, respectively) and no significant deviation from normal distribution for patients with a surgical diagnosis of DIE (*p* = 0.589 and *p* = 0.172, respectively). The analysis of the differences of AGD measurements depending on surgical findings showed no statistically significant results, both when looking at the mean values formed from the measurements of the two readers and when looking at the individual measurements of readers 1 and 2 (Table 2).

**Table 2:** Comparison of anogenital distances in millimeters with standard deviations in patients with and without a surgical diagnosis of DIE, peritoneal endometriosis with and without DIE, and ovarian endometriosis with *p*-values of Wilcoxon test.

	Surgery								
	DIE			Per. endometriosis w/wo DIE			Ovarian endometriosis		
	+ (*n* = 67)	− (*n* = 60)	*p*	+ (*n* = 85)	− (*n* = 42)	*p*	+ (*n* = 31)	− (*n* = 96)	*p*
MRI-AGD-AC, mean, readers 1 + 2	76.5 ± 9.2	75.6 ± 10.7	0.413	75.5 ± 9.3	77.1 ± 11.2	0.641	78.0 ± 9.7	75.4 ± 10.0	0.155
MRI-AGD-AF, mean, readers 1 + 2	33.7 ± 5.3	32.5 ± 4.5	0.110	33.4 ± 5.1	32.7 ± 4.7	0.323	34.1 ± 5.7	32.9 ± 4.7	0.150
MRI-AGD-AC, reader 1	75.1 ± 9.6	74.2 ± 11.3	0.374	74.2 ± 9.7	75.6 ± 11.9	0.731	76.6 ± 10.5	74.1 ± 10.4	0.221
MRI-AGD-AF, reader 1	31.7 ± 6.2	30.9 ± 5.0	0.323	31.6 ± 5.9	30.8 ± 5.0	0.433	32.1 ± 6.6	31.1 ± 5.3	0.161
MRI-AGD-AC, reader 2	77.7 ± 9.6	76.9 ± 10.6	0.530	76.8 ± 9.5	78.5 ± 11.2	0.486	79.4 ± 9.7	76.7 ± 10.1	0.137
MRI-AGD-AF, reader 2	35.7 ± 5.6	34.1 ± 5.0	0.082	35.1 ± 5.3	34.5 ± 5.4	0.436	36.0 ± 5.9	34.6 ± 5.2	0.200

DIE, deep-infiltrating endometriosis; Per., peritoneal; w/wo, with and without.

A comparison of the AGDs only for the measurements with good measurability noted by both readers (*n* = 53) also revealed no statistically significant differences in the individual patient groups (Table 3).

**Table 3:** Comparison of anogenital distances with good measurability noted by both readers in millimeters with standard deviations in patients with and without a surgical diagnosis of DIE, peritoneal endometriosis with and without DIE, and ovarian endometriosis with *p*-values of Wilcoxon test.

	Surgery								
	DIE			Per. endometriosis w/wo DIE			Ovarian endometriosis		
	+ (*n* = 28)	− (*n* = 25)	*p*	+ (*n* = 34)	− (*n* = 19)	*p*	+ (*n* = 11)	− (*n* = 42)	*p*
MRI-AGD-AC, mean, readers 1 + 2	77.7 ± 10.4	77.3 ± 12.4	0.656	76.9 ± 10.5	78.6 ± 12.8	0.767	82.2 ± 10.9	76.2 ± 11.1	0.109
MRI-AGD-AF, mean, readers 1 + 2	34.8 ± 6.0	32.4 ± 3.4	0.084	34.2 ± 5.6	32.6 ± 3.8	0.274	35.4 ± 7.2	33.2 ± 4.3	0.223
MRI-AGD-AC, reader 1	76.3 ± 10.5	76.4 ± 12.4	0.618	75.6 ± 10.6	77.6 ± 12.6	0.882	81.4 ± 11.1	75.1 ± 11.1	0.109
MRI-AGD-AF, reader 1	33.2 ± 6.4	31.2 ± 3.7	0.159	32.8 ± 6.0	31.2 ± 3.8	0.290	33.6 ± 7.4	31.9 ± 4.7	0.223
MRI-AGD-AC, reader 2	79.0 ± 10.5	78.1 ± 12.7	0.569	78.0 ± 10.6	79.6 ± 13.3	0.838	83.3 ± 11.0	77.3 ± 11.5	0.109
MRI-AGD-AF, reader 2	36.3 ± 6.6	33.6 ± 4.5	0.092	35.6 ± 6.2	34.0 ± 5.1	0.308	37.2 ± 8.3	34.4 ± 5.0	0.199

DIE, deep-infiltrating endometriosis; Per., peritoneal; w/wo, with and without.

**Fig. 4:**
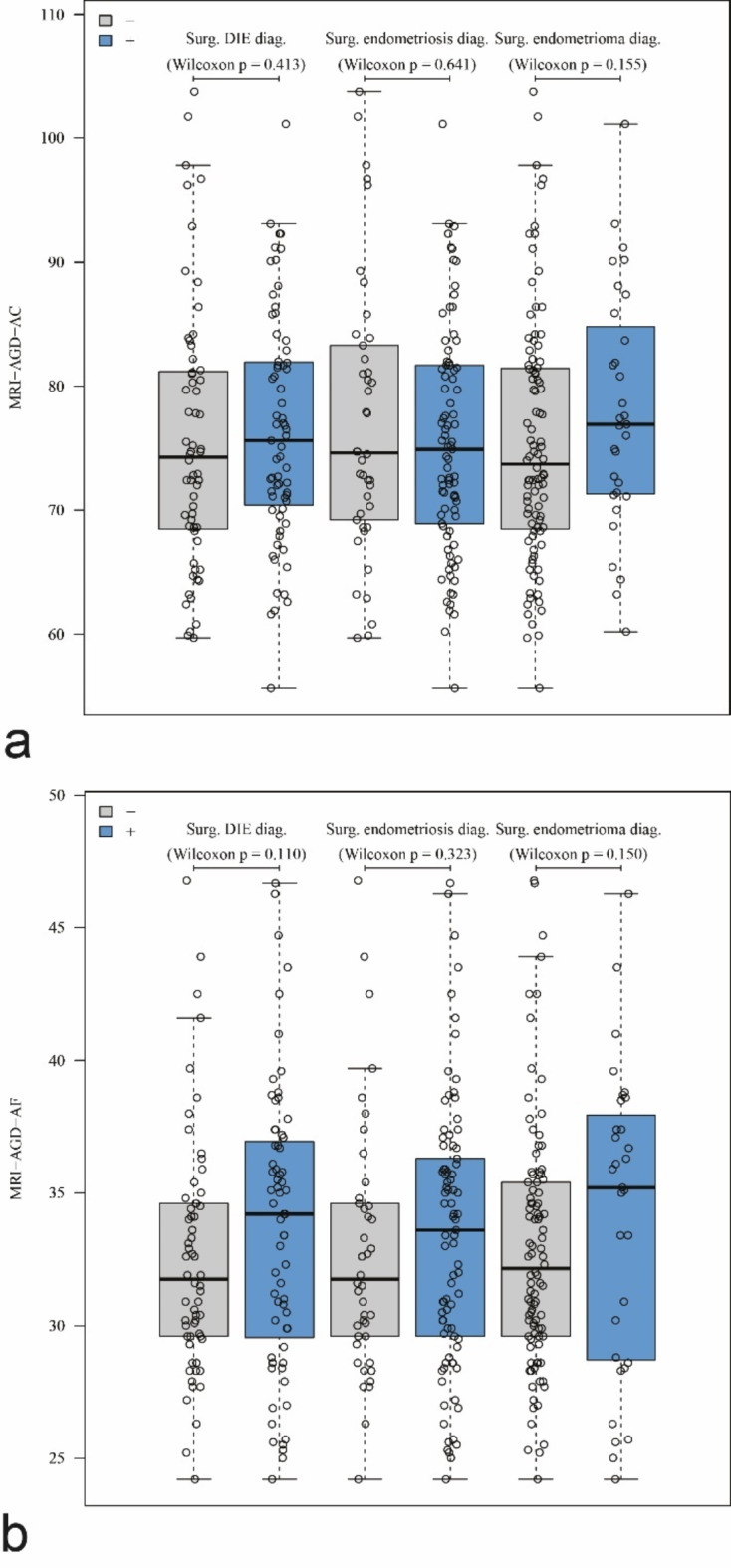
Distribution of the MRI-AGD-AC (**a**) and the MRI-AGD-AF (**b**) values depending on the surgical diagnosis of DIE, peritoneal endometriosis with and without DIE, and ovarian endometriosis. The boxes represent the values for the median and the 25th and 75th percentiles. The whisker plots represent the maximum and minimum values, excluding outliers.

The average AGD measurements and their distribution within patient groups are summarized in Fig. 4.

Results of the binary logistic regression models for the variables “DIE”, “peritoneal endometriosis with and without DIE”, and “ovarian endometriosis” are shown in Table 4.

**Table 4:** Comparison of ordinary least squares (OLS) and logistic (Logit) regressions for the variables “DIE”, “peritoneal endometriosis with and without DIE”, and “ovarian endometriosis”.

	Surgery					
	DIE		Per. endometriosis w/wo DIE		Ovarian endometriosis	
	OLS 1a	Logit 1b	OLS 2a	Logit 2b	OLS 3a	Logit 3b
Age	0.003 (*p* = 0.654)	0.013 (*p* = 0.635)	−0.007 (*p* = 0.311)	−0.029 (*p* = 0.323)	0.002 (*p* = 0.681)	0.013 (*p* = 0.685)
BMI	0.017 (*p* = 0.067)	0.077 (*p* = 0.072)	0.005 (*p* = 0.539)	0.024 (*p* = 0.548)	0.007 (*p* = 0.420)	0.031 (*p* = 0.441)
MRI-AGD-AC, readers 1 + 2	−0.010 (*p* = 0.152)	−0.043 (*p* = 0.142)	−0.013 (*p* = 0.063)	−0.056 (*p* = 0.067)	0.001 (*p* = 0.882)	0.006 (*p* = 0.865)
MRI-AGD-AF, readers 1 + 2	0.017 (*p* = 0.162)	0.073 (*p* = 0.163)	0.022 (*p* = 0.060)	0.101 (*p* = 0.065)	0.004 (*p* = 0.693)	0.023 (*p* = 0.686)
n	127	127	127	127	127	127
AIC	189.8	179.4	175.8	166.3	155.0	148.5
BIC	206.9	193.6	192.9	180.6	172.1	162.8
Log.-Lik.	−88.912	−84.702	−81.923	−78.167	−71.504	−69.273
F value	1.511	1.403	1.235	1.168	0.672	0.661

DIE, deep-infiltrating endometriosis; Per., peritoneal; w/wo, with and without; OLS, Ordinary least squares regression; Logit, Logistic regression; AIC, Akaike-Information-Criterion; BIC, Bayesian-Information-Criterion; Log.-Lik., Log-likelihood.

**Fig. 5:**
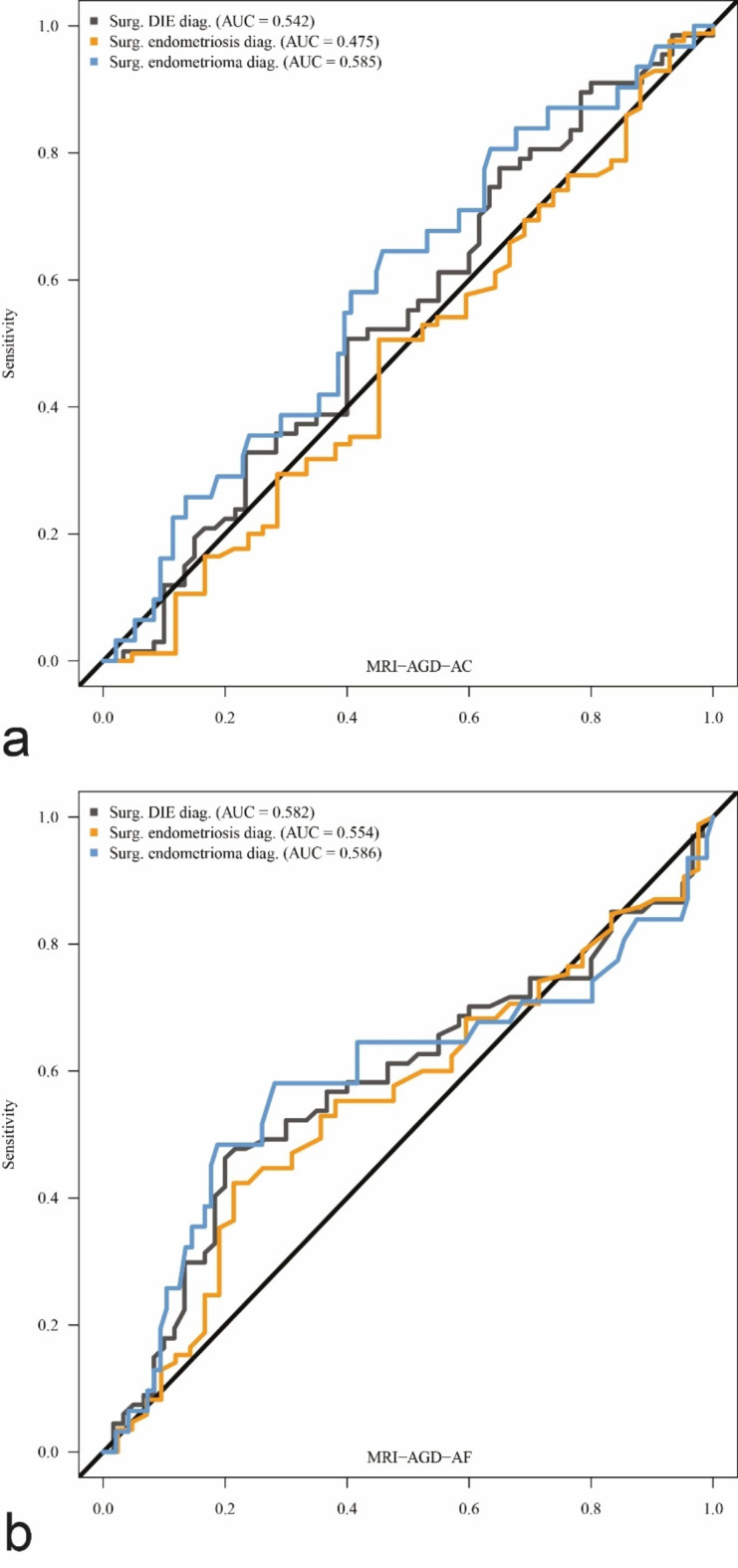
ROC Curves of MRI-AGD-AC (**a**) and MRI-AGD-AF (**b**) for deep infiltrating endometriosis (DIE), peritoneal endometriosis with and without DIE, and ovarian endometriosis.

ROC curves are presented in Fig. 5.

The AUC of MRI-AGD-AC was 0.542 (95% CI: 0.441–0.644; *p* = 0.415) for DIE, 0.475 (95% CI: 0.365–0.584; *p* = 0.648) for peritoneal endometriosis with and without DIE, and 0.585 (95% CI: 0.472–0.698; *p* = 0.140) for ovarian endometriosis. The AUC of MRI-AGD-AF was 0.582 (95% CI: 0.482–0.683 *p* = 0.109) for DIE, 0.554 (95% CI: 0.450–0.658; *p* = 0.310) for peritoneal endometriosis with and without DIE, and 0.586 (95% CI: 0.454–0.718; *p* = 0.200) for ovarian endometriosis.

## Discussion

This study aimed to evaluate measurements of MRI-AGD-AC and MRI-AGD-AF for the prediction of endometriosis in patients without prior abdominal surgeries, examined for endometriosis on MRI, and subsequently undergoing surgery within one year of the MRI examination. Our findings did not indicate a statistically significant difference in the averaged AGD measurements of two readers between patients with and without a surgical diagnosis of DIE (*p* = 0.110 to *p* = 0.413), between patients with and without a surgical diagnosis of peritoneal endometriosis with and without DIE (*p* = 0.323 to *p* = 0.641), and between patients with and without a surgical diagnosis of ovarian endometriosis (*p* = 0.150 to *p* = 0.155). Also, no statistically significant differences were found between these patient groups when only the measurements of the more experienced reader were considered (*p* = 0.161 to *p* = 0.731).

No data are available to date on the interobserver agreement of MRI-based AGD measurements. The applicability of AGD measurements on MRI in our study was good, with ICC analysis showing better agreement between the two readers in the measurement of MRI-AGD-AC (ICC 0.92; 95% CI: 0.83–0.95) than in the measurement of MRI-AGD-AF (ICC 0.68; 95% CI: 0.27–0.83). This is probably due to the less distinct definition of the posterior fourchette in the MRI images, whereas the anatomical landmarks of the MRI-AGD-AC are more often clearly identifiable. In the only available study in which AGD measurements were performed in MRI by Crestani et al., the measurements were performed by a single radiologist, and the reproducibility of the measurements was not investigated^14^. Our data favor the measurement of MRI-AGD-AC over MRI-AGD-AF as a more robust and easily reproducible parameter, even for inexperienced readers (e.g., medical students following a training measure, as in our study).

In general, and perhaps unsurprisingly, the more experienced reader rated the measurability more favorably than the inexperienced reader (good measurability in 73.2% and 44.9%, respectively, *p* < 0.001). For the AGD measurements for which good measurability was documented by both readers (*n* = 53), the agreement was slightly better than in the entire collective (increase in ICC for MRI-AGD-AC from 0.92 to 0.96 and for MRI-AGD-AF from 0.68 to 0.73). As these differences are only minor, this confirms that AGD measurements on MRI are possible for inexperienced readers despite the perceived difficulty in measurements compared to more experienced readers.

The challenging question arises as to why our results cannot confirm the results of several other studies on AGD^3,10–14,19,21^, which found some degree of association between AGD and endometriosis. Crestani et al. reported an AUC of 0.840 (95% CI: 0.782–0.898) for AGD-AF and an AUC of 0.756 (95% CI: 0.684–0.828) for AGD-AC for predicting the presence of endometriosis^13^. Sánchez-Ferrer et al. reported an AUC of 0.91 (95% CI: 0.84–0.97) for AGD-AF and an AUC of 0.64 (95% CI: 0.53–0.75) for AGD-AC for predicting the presence of DIE^11^. In contrast to our study, other groups examined different collectives with possible confounders. The control groups did not correspond to the collective for which AGD measurements to predict endometriosis would be applied. In the study by Crestani et al., surgeries in the control group were performed for various reasons (e.g., hysterectomy for leiomyomas, salpingo-oophorectomy for ovarian lesions, etc.), but not to diagnose and possibly treat endometriosis^13^. The specification of our study population, in contrast, is that it represents the population for which the use of a biomarker for endometriosis would be reasonable—i.e., patients with clinically suspected endometriosis who have not yet undergone surgery and in whom endometriosis is therefore not yet confirmed. In the study by Buggio et al., in which care was taken to avoid confounders through the selection criteria, no correlation was found between clinically measured AGD and the presence of endometriosis, as in our study^15^.

There is heterogeneity in the execution of AGD measurements between different groups. In the only study in which AGD measurements were performed on MRI by Crestani et al., it is stated that measurements were taken from the anterior edge of the anal canal to the posterior wall of the clitoris/vagina, but no MR images are presented^14^. Mean values of 40.4 mm ± 7.3 for MRI-AGD-AC and 13.3 mm ± 3.9 for MRI-AGD-AF were reported for the endometriosis group, i.e., substantially shorter distances than in our investigation (75.5 mm ± 9.3 and 33.4 mm ± 5.1, respectively). For clinical measurements of AGD, Buggio et al. reported mean values of 76.0 mm ± 12.1 for AGD-AC and 22.8 mm ± 5.0 for AGD-AF in a patient group affected by DIE^15^. Thus, the values for AGD-AC were similar to those found in our study, but the values for AGD-AF were substantially lower. We decided to perform the AGD measurements on MRI from the center of the lower edge of the anal canal (i.e., the anus) due to the favorable reproducibility, like Liu et al. described for clinical AGD measurements^19^. The good to excellent agreement between two readers for MRI-AGD-AC in our study documents the reliability of this approach, whereas, in the measurement of MRI-AGD-AF, a lower reliability was shown possibly due to the less clearly defined posterior fourchette.

It has been discussed that pathophysiologically, a short AGD in women with endometriosis indicates an early exposure to genetic and epigenetic factors and endocrine disruptors during intrauterine life^13^. Various other diseases have been identified that show a relationship to AGD, such as prostate cancer, PCOS, pelvic organ prolapse (POP), hypospadias, testicular germ cell tumors, testicular microlithiasis, and azoospermia^3,22–24^. Our results do not provide a favorable answer regarding the value of AGD measurements on MRI for predicting endometriosis. Nevertheless, MRI is a valuable non-invasive diagnostic tool for detecting and determining the extent of endometriosis^4,5^.

Numerous biomarkers for endometriosis are currently the subject of research, including glycoproteins, inflammatory cytokines, apoptosis markers, immunological markers, angiogenesis markers, hormone markers, miRNAs, neuropeptide markers, proteomics, metabolomics, genomics, and the microbiome^7,25,26^. A robust and reliable non-invasive marker for the diagnosis of endometriosis has not yet been identified^27,28^. A questionnaire-based approach has recently shown promising results with a sensitivity of 90.4% and a PPV of 87.4%^29^. Given the frequency and significance of the disease, which has a substantial negative impact on patients’ quality of life, further efforts to identify non-invasive diagnostic markers of endometriosis and to facilitate effective and early treatment are of great interest^30^.

There are limitations to our study. The study was conducted retrospectively at a single center. We have used surgery as the gold standard, although limitations of laparoscopy are known^31^, such as in the visual identification of lesions with the conventional white light technique^32^. Therefore, we cannot completely rule out cases in the control groups (no surgical detection of DIE, peritoneal endometriosis with and without DIE, or ovarian endometriosis). On the other hand, surgical exclusion of endometriosis should be regarded as a high-quality criterion, even if there is an increasing restraint in the indication of surgery for endometriosis in favor of non-invasive diagnostic and therapeutic approaches^33^.

In conclusion, our results do not indicate that anogenital distance, measured on MRI, is a valuable biomarker for patients with clinically suspected endometriosis. No association of MRI-AGD with deep infiltrating endometriosis, peritoneal endometriosis with and without DIE, or ovarian endometriosis was demonstrated.

## Data Availability

The datasets generated during and analysed during the current study are available from the corresponding author on reasonable request.
